# Around the Hospitals

**Published:** 1893-10-28

**Authors:** 


### Around the Hospitals.

Notice to the Managers of Institutions.—Special space is now reserved for the insertion of notices of hospital meetings and festivals. The Editor requests that all notices may reach The Hospital Office, 428, Strand, at least one week before the date of each meeting.

**The Northampton General Infirmary.**—The whole tenure of the annual meeting of the Northampton General Infirmary showed a spirit of progress and enlightenment which augurs well for the future prospects of the institution, and affords good reason for the present satisfactory condition. In reporting the improvements which have recently been effected, the management still frankly admit that their hospital is far from complete in many ways, and in pointing out where improvement is desirable or imperative, they place the public in the position of responsibility which they will doubtless not fail to accept. It is now one hundred years since the infirmary was removed to the present site. Its capacity originally was but for 96 beds. During the past year the daily average of beds occupied has been just 147. The additions that have been made from time to time has consisted in a board-room, with wards along it, extension of the south wing, the building of rooms between the north wings, an out-patients' department, and the Victoria wing; whilst during the last two years the north-east wing has been rebuilt and offices have been reconstructed. The management now point out that the operation-room is quite insufficient for so large an infirmary, and that a better accident room and waiting-room are much needed. Besides this, a new warming system should be adopted and external repairs carried out. It could be wished that some friend of the infirmary should take up the matter, and render the infirmary so complete and efficient as to be a credit to the large neighbourhood which it serves. Last year the income showed a slight decrease, but the institution was at the same time in receipt of the largest legacy which has as yet fallen to its share. The work of the infirmary shows a steady tendency to increase, and the efforts of the Secretary (Mr. Vernon) and of the President (Mr. Mansfield) are severely taxed to maintain an adequate income. At the annual meeting Canon Hull spoke very strongly against the system of letters of recommendation for patients.

**The Southampton Free Eye and Ear Hospital.**—The establishment of this hospital has been attended with great success. It has been erected only four years, and is already a recognised special ophthalmic hospital. The present site, in a crowded street, is felt to be very unsuitable, and the accommodation is already too limited. The success of the institution has encouraged the management to seek for other quarters, and Mr. Garton has offered to present a site. Subscriptions towards the building, amounting to £850, have already been received.

